# Accuracy and Precision of the Subjective Visual Vertical According to Age in Adults With/Without Diabetes

**DOI:** 10.3390/diagnostics16142144

**Published:** 2026-07-08

**Authors:** Kathrine Jáuregui-Renaud, José Adán Miguel-Puga, Aida García-López, María de Lourdes Tirado-Mondragón

**Affiliations:** 1 Unidad de Investigación Médica en Otoneurología, Instituto Mexicano del Seguro Social, Mexico City 06720, Mexico; adan.miguel@imss.gob.mx (J.A.M.-P.); draidagarcia@gmail.com (A.G.-L.); 2 Hospital General de Zona 8, Instituto Mexicano del Seguro Social, Mexico City 01090, Mexico; marilutm16@hotmail.com

**Keywords:** vestibular, subjective visual vertical, graviception, aging, diabetes, physical activity, sitting time

## Abstract

**Background/Objectives**: Multisensory inputs generate a common gravity-reference frame, but just the otoliths sense the gravity vector. Graviception is frequently assessed by setting a luminous line to the subjective visual vertical (S.V.V.). Population aging and diabetes prevalence, with insufficient physical activity, imply the need to ponder these factors in clinical assessments. This study aimed to assess S.V.V. accuracy/precision in adults with/without diabetes, according to age, physical activity, and general characteristics. **Methods**: Participants were 262 adults without diabetes (21–80 years old (y.o.)) and 187 adults with diabetes (28–80 y.o.; matched with 187 without diabetes). All participants had no history of otology/vestibular/neurology/autoimmune/orthopedic/severe renal disease or proliferative retinopathy or traumatic injury or balance complaints. After audiology–vestibular evaluations, the International Physical Activity Questionnaire was self-administered and the S.V.V. was estimated during static and on-axis rotation conditions. **Results**: In participants without diabetes, S.V.V. precision but not S.V.V. accuracy decreased after the age of 50 years, with no further decrease after the age of 70 years (up to 80 y.o.); the main cofactors contributing to the variability on the S.V.V. precision were physical activity and sitting time, with inconsistent contribution from a history of COVID-19 (R = 0.44 static and 0.35 on-axis, *p* < 0.00001). In participants with diabetes, the major contribution to the S.V.V. precision variability was from diabetes and age (R = 0.33, static and 31 on-axis, *p* < 0.00001). **Conclusions**: Apart from aging, the sensorimotor process affected by insufficient physical activity and sedentary behavior includes vestibular decline. The complex pathophysiology of diabetes may account for the contribution from these cofactors.

## 1. Introduction

To interact with the surroundings, adequate motor control and spatial orientation require perception of the head–body posture relative to gravity. Vestibular, visual, and body information generate a common gravity-reference frame [1]. The relative weight of each sensory signal depends on the specific task, while just the otolith maculae directly sense the gravity force vector [2].

Evidence supports that Bayesian causal inference may drive integration of signals of variable reliabilities [3]. Predictive coding and active inference theory suggests the construction of estimates of the sensorimotor transformations to optimally adapt to environment demands [4,5]. However, updates to an active internal model may be a function of the quality of the evidence upon which it is built and the subsequent errors. Then, any estimation of sensory inputs would require both closeness to the real value (accuracy) and consistency across estimations (precision). In the case of incoherent sensory inputs, consistent discrepancies may be reduced by recalibration [6]. Conversely, fluctuating discrepancies may be lessened by multisensory integration; the more reliable (precise) signal would be taken as the more veridical one [7].

A proxy of the perception of the head posture relative to gravity is to set a luminous line to the Subjective Visual Vertical (S.V.V.); in an upright position with no visual cues, healthy human beings can accurately indicate the perceived vertical within the range of veracity of ±2° [8]. Assessment of the S.V.V. can be performed in a variety of conditions, including static (head upright/tilted) with greater vestibular weight while upright [9], or during yaw rotation with horizontal canal stimulation, with either bilateral or unilateral utricular stimulation by on-axis or off-axis rotation (to provoke asymmetry of the utricular inputs) respectively [10]. The accuracy of the S.V.V. estimation can be evaluated by the mean deviation error of repeated estimations using the average, while the precision of the S.V.V. estimation can be assessed by the variability of repeated estimations using the standard deviation [11]. In an upright position, in the dark, S.V.V. precision is regulated by the otolith organs and by central computational mechanisms, which maximize the performance of verticality estimates in this position [12,13]. During estimations at different tilt angles, visual information may improve the accuracy while the precision remains unchanged [13], supporting that accuracy and precision are not dependent on each other [14].

Apart from the measurement technique [15], a variety of factors may account for variability in S.V.V. estimation, including age. Compared to young adults, old adults may show decreased [16,17,18] or similar S.V.V. accuracy [15,19,20,21]; while aging may decrease S.V.V. precision [17,18], with no difference by sex [15,16,17,18,19,21].

In addition, a variety of cofactors may modify the use of sensory signals for head–body balance, including disease and physical activity [22,23]. Adults with type 2 diabetes (diabetes) may have impairment of the otolith organs [24,25,26] showing decreased S.V.V. accuracy in both static and during on- and off-axis rotation while in an upright position [26]. In contrast, regular practice of physical activity can reduce the interference of inadequate vestibular signals on postural control, suggesting faster switching among sensory inputs [22,27]; additionally, sitting behavior also requires attention due to contemporary lifestyle patterns [28].

Currently, aging of the general population [29], with an increasing global prevalence of diabetes [30] and insufficient physical activity with sitting time increase [31] imply the need to ponder these cofactors for an adequate interpretation of head-body balance assessments. A general hypothesis is that aging and diabetes may have a negative impact on S.V.V. accuracy/precision that could be ameliorated by physical activity. However, more clinical information is required, including studies on the accuracy/precision of the S.V.V. in adults with diabetes. The aim of this study was to assess the S.V.V. accuracy/precision in adults with/without type 2 diabetes (diabetes), according to age, physical activity, and general characteristics.

## 2. Materials and Methods

The study was conducted in accordance with the Declaration of Helsinki and its amendments. The research protocol was approved by the Institutional Research and Ethics Committees of Instituto Mexicano del Seguro Social (IMSS R2018-785-046, 29 May 2018). All participants provided their written informed consent to participate in the study.

### 2.1. Participants

They were

- 262 participants without diabetes aged 21 to 80 years old (123 females and 139 males).
- 187 participants with diabetes aged 28 to 80 years (86 females and 101 males), who were age matched for statistical analysis with 187 participants without diabetes who were selected among all the participants without diabetes, aged 28 to 80 years (106 females and 81 males).

Consecutive recruitment of all participants was performed in a healthcare institution, according to the following selection criteria: no history of otology/vestibular/autoimmune/orthopedic/severe renal disease or proliferative retinopathy or traumatic injury or neurology disease apart from peripheral polyneuropathy. One more participant without diabetes and three participants with diabetes did not complete the study protocol due to claustrophobia during the audio-vestibular assessments.

A sample size of 185 participants was calculated to estimate the mean accuracy of the S.V.V., considering internal reference data from 105 adults (aged 19–83 years) who made estimates with a standard deviation of 0.5°. The calculation of the sample size was performed using a 0.1° error, with a 99% confidence interval and 10% dropouts [32].

### 2.2. Procedures

- Preliminary evaluations To ascertain the selection criteria, in all participants, evaluations included medical history, otology (otoscopy and tympanometry), audiology and oculomotor assessments. Vestibular evaluation included sinusoidal harmonic acceleration in the dark at both 0.16 and 1.28 Hz (60°/s peak velocity) (I-Portal-NOCT-Professional, Neuro-Kinetics, Pittsburgh, PA, USA). In all participants, clinical tests for peripheral neuropathy were performed; additionally, in participants with diabetes, specialized assessments were performed on visual acuity with retinoscopy and electromyography.
- Questionnaires Self-administration of an in-house general health questionnaire (including alcohol/tobacco use), and the short form of the International Physical Activity Questionnaire [28], which provides information on both physical activity and sitting time. According to Version 2.0 Guidelines, responses were used to estimate three levels of physical activity (low/moderate/high) (https://ugc.futurelearn.com/uploads/files/bc/c5/bcc53b14-ec1e-4d90-88e3-1568682f32ae/IPAQ_PDF.pdf) (accessed on 29 June 2026) [33].

Subjective Visual Vertical Assessment was performed inside a circular, lightproof booth, in a seated upright posture (1 m from the enclosure). Participants were provided with right and left handles to control line images of 35 cm, using randomly selected positive/negative preset angles. After standardized instructions and practice, at least 10 S.V.V. estimations were recorded during each of two conditions: static and on-axis constant yaw rotation at 300°/s (I-Portal-NOCT-Professional, Neuro-Kinetics, Pittsburgh, PA, USA). The accuracy was assessed by the average and the precision by the standard deviation of each individual set of S.V.V. estimations.

### 2.3. Statistical Analysis

Data distribution was assessed by Kolmogorov–Smirnov test. Exploratory bivariate analysis was performed according to data distribution, either by “*t*” test, Pearson’s correlation coefficient and analysis of variance, or by Mann–Whitney “U” test, Spearman’s correlation coefficient and Kruskal–Wallis test. Comparisons between proportions were performed using Z test. According to the results of the bivariate analysis, multivariate analyses of covariance were performed on two data sets: 1. all the participants without diabetes (aged 21 to 80 years; N = 262) and 2. the participants with diabetes (aged 28 to 80 years, N = 187) and the age-matched participants without diabetes (aged 28 to 80 years, N = 187). The significance level was set at 0.05.

## 3. Results

### 3.1. Participants Without Diabetes

The majority of participants had a body mass index ≥ 25, while the report of tobacco use was not frequent and alcohol use (with no abuse) was reported by 30% of the participants, mainly males. Almost half of the participants had a history of COVID-19 (hospitalization < 5%); other comorbidities were less frequent (Table 1). High physical activity was reported by almost two thirds of the participants, while the sitting time was variable (Table 1).

#### 3.1.1. Bivariate Analysis

Sinusoidal rotation in the dark at 0.16 Hz showed decreasing vestibulo-ocular reflex gain after the age of 50 years, with no decline at 1.28 Hz (Table 2).

**Figure 1:**
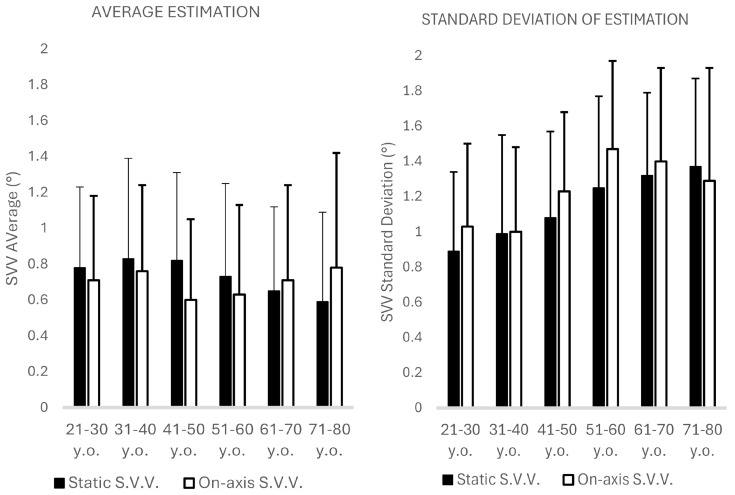
Mean and standard deviation of the accuracy and precision of the Subjective Visual Vertical (S.V.V.) during static and on-axis estimation of 262 adults by decade of life.

The S.V.V. accuracy showed no asymmetric responses, and no statistical difference was observed during the two conditions, static and on-axis (Table 2, Figure 1). However, S.V.V. precision decreased after the age of 50 years in the two conditions, without further decrease in participants > 70 years old (Table 2, Figure 1). Low and moderate linear correlations were observed between the age and the following variables: the vestibulo-ocular reflex gain in the dark at 0.16 Hz (Pearson’s r = −0.28, *p* < 0.00001), the sitting time (Pearson’s r = −0.31, *p* < 0.00001), the S.V.V. accuracy in the static condition (Pearson’s r = −0.13, *p* = 0.03), the S.V.V. precision in the static condition (Pearson’s r = 0.40, *p* < 0.00001) and the S.V.V. precision in the on-axis condition (Pearson’s r = 0.26, *p* = 0.00001).

#### 3.1.2. Analysis of Covariance

The results of the multivariate analysis are shown in Table 3. The study variables contributing to the variability in S.V.V. precision in the two study conditions were the age of the participants with beta values of 0.33 (95% Confidence Interval (C.I.) 0.22–0.45) for the static condition and 0.21 (95% C.I. 0.09–0.33) for the on-axis condition; the sitting time with beta values of −0.12 (95% C.I. −0.24–−0.01) for the static condition and −0.14 (95% C.I. −0.26–−0.01) for the on-axis condition; the physical activity category with beta values of −0.12 (95% C.I. −0.23–−0.01) for the static condition and −0.19 (95% C.I. −0.30–−0.07) for the on-axis condition; and a borderline contribution of the history of COVID-19, just for the static condition (beta 0.10, 95% C.I. −0.001–0.21).

### 3.2. Participants with/Without Diabetes

The majority of participants with/without diabetes had a body mass index ≥ 25; tobacco use and alcohol use were reported by ≤30% of the participants, mainly males (Table 4). The majority of participants with diabetes had peripheral neuropathy (73.7%, 95% C.I. 67.3–80.0%) and half of them had retinopathy (43.5%, 95% C.I. 36.3–50.6%). The general characteristics of the participants without diabetes were similar to those observed in the whole group of participants without diabetes.

#### 3.2.1. Bivariate Analysis

Corrected refraction errors were more frequent among participants without diabetes than among those with diabetes (Z = 3.85, *p* = 0.0002). In the two groups, almost half of all the participants had a history of COVID-19 (hospitalization < 5%), with no difference between the groups. Systemic high blood pressure was more frequent in participants with diabetes than in those without diabetes (Z = 2.94, *p* = 0.003), as well as dyslipidemia (Z = 2.94, *p* = 0.003) (Table 4). Almost two thirds of the participants without diabetes reported high physical activity, while less than half of the participants with diabetes reported high physical activity (Z = 3.62, *p* = 0.0002); the sitting time was variable in the two groups.

**Figure 2:**
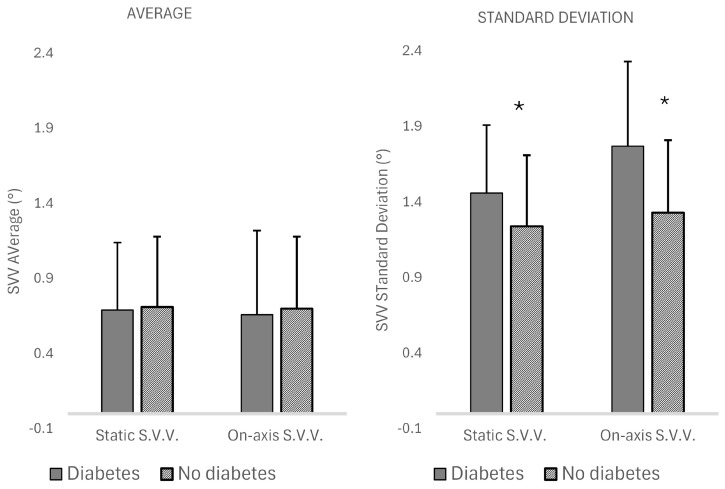
Mean and Standard Deviation of the accuracy and precision of the Subjective Visual Vertical (S.V.V.) during static and on-axis estimation of 187 adults with diabetes and 187 without diabetes. Statistical differences are marked using *.

Comparison between the participants with/without diabetes showed difference on the vestibulo-ocular reflex gain in the dark at 0.16 Hz (Table 5). The S.V.V. accuracy showed no asymmetric responses and the absolute values were similar in the two groups, during the two conditions, static and on-axis (Table 5, Figure 2). However, participants with diabetes exhibited decreased S.V.V. precision in the two conditions (Table 5, Figure 2).

#### 3.2.2. Analysis of Covariance

The results of the multivariate analysis are shown in Table 6. The study variables contributing to the variability in S.V.V. precision in the two study conditions were the age of the participants with beta values of 0.23 (95% C.I. 0.13–0.32) for the static condition and 0.16 (95% C.I. 0.06–0.26) for the on-axis condition; diabetes with beta values of −0.21 (95% C.I. −0.31–−0.11) for the static condition and −0.26 (95% C.I. −36–−0.16) for the on-axis condition; and the vestibulo-ocular gain at 1.28 Hz just for the static condition (beta −0.13, 95% C.I. −0.23–−0.03). No other contributions were evident in this analysis, including those contributing to the variance of the S.V.V. precision in all the participants without diabetes.

## 4. Discussion

The aim of this study was to assess the S.V.V. accuracy and precision in adults with/without diabetes, according to age, physical activity, and general characteristics. In participants without diabetes, and no history of otology or vestibular or neurology disease or balance complaints, the S.V.V. precision but not the S.V.V. accuracy decreased after the age of 50 years, with no further decrease after the age of 70 years (up to 80 y.o.). The main cofactors contributing to the variability among participants without diabetes on the S.V.V. precision were physical activity and sitting time, with inconsistent contribution from a history of COVID-19. In participants with diabetes and age-matched participants without diabetes, the major contribution to the S.V.V. precision variability was from diabetes and age. The results were similar during the two conditions, with/without otolith and horizontal semicircular canal stimulation. Of note, the whole model correlations obtained by the multivariate analysis were moderate; then, cofactors not included in this study, such as cognitive resources, could have contributed to the S.V.V. precision variability.

### 4.1. Age

In participants without diabetes, the decrease in the vestibulo-ocular reflex gain to sinusoidal rotation at 0.16 Hz related to aging was consistent with previous studies. In 216 individuals from 7 to 81 years old, increasing age was related to amplitude decrease with less compensatory response phase to rotation [34]. Over a follow-up of five years, 57 individuals (mean age 82 years) showed amplitude-dependent decrease in gain and increase in phase lead during low-frequency sinusoidal rotation, as well as shortening of the vestibulo-ocular response time constant to step changes in angular velocity [35]. Additionally, in this study we also observed age-related S.V.V. precision decrease with aging.

At varying times, structures relevant for adequate vestibular function may degenerate with age [36,37,38]. Decline for total, type I, and type II hair cell densities in the five vestibular sense organs were evident in 67 temporal bones of normal individuals from birth to 100 years old (y.o.) [36]. Consistently, vestibular nerve fibers may decrease with increasing age [37]. Additionally, in mice, synaptopathy can be associated with late onset dysfunction of the otolith organs [38]. In 80 individuals aged ≥60 years, there was association between reduced vestibular function and reduced volumes of the hippocampus and the entorhinal cortex [39]. The vestibular function was also related to the shape of the hippocampus, amygdala, thalamus, caudate nucleus, putamen, and entorhinal cortex-trans-entorhinal cortex complex [39]. In patients with acute unilateral isolated infarcts in the thalamus, specific thalamic subnuclei were associated with either ipsiversive or contraversive deviations of the perception of verticality [40].

The estimation of S.V.V. also requires a wide cortical network, and effective integration of sensory information. In adults, neural activation spreads to the temporo-occipital and parieto-occipital cortices, with a right dominance tendency, and also to the cerebellum and the brainstem [41]. During S.V.V. estimation, both old and young adults may display activation of the right supramarginal gyrus and the left dorsolateral superior frontal gyrus, while just old adults may exhibit additional activation in the bilateral postcentral gyrus and the right middle frontal gyrus. Old adults may also have bilateral dominance across sensory, dorsolateral prefrontal and motor cortices. In contrast, young adults may exhibit right lateralization in sensory and dorsolateral prefrontal cortices, and left lateralization in the motor cortex [17].

In this study, the finding of S.V.V. precision decrease in mature adults with no further decay after the age of 70 years (up to 80 y.o.) is consistent with the evidence in healthy individuals of an inverse U-shaped curve for the static ocular torsion and torsional nystagmus responses to binaural and monaural galvanic vestibular stimulation according to age [42].

Several hypotheses have been suggested to understand compensation for the vestibular decline with aging. Since deterioration of the peripheral organ may precede neural and ganglionic deterioration, compensatory increased sensitivity of afferent nerve fibers or central mechanisms could maintain function despite reduced peripheral input [42]. Central mechanisms may reweigh sensory information according to their relative noise; high visual dependence in the elderly can be associated with decreased static balance under conditions of limited visual and somatosensory inputs [43]. Age-dependent functional connectivity decrease with variability increase may be related to reciprocal cortico-cortical inhibition deterioration with age and multimodal vestibular integration of sensory inputs [44].

**Figure 3:**
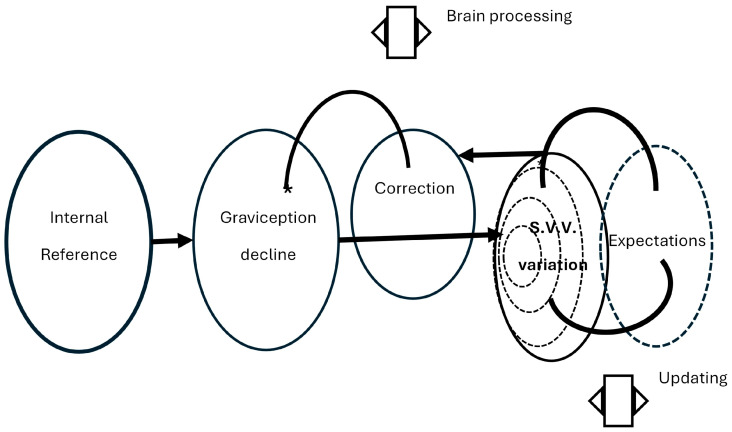
In the context of the predictive coding and action inference theory [4,5], sensory input decline may provoke increasing variation in the discrepancy between the expected and the observed information, hampering updating of expectations by recalibration; then, measurement accuracy could be corrected mainly by multisensory processing [46].

In a statistically optimal way, Bayesian optimal adaptation can explain age-dependent changes in the vestibulo-ocular reflex in response to age-dependent sensory anatomical changes over many years [45]. In the context of the theory of predictive coding and active inference [4,5], the results of this study support the proposal that once sensory misinformation disrupts the physiological processes supported by the sensory input, the variability of the discrepancy between predictions and observations may interfere with adaptation through updating expectations based on experience, while brain processes could provide corrections to maintain function (Figure 3). However, this study supports that internal or external cofactors, such as individual threats or environmental context, can disrupt these processes.

### 4.2. Physical Activity and Sitting Time

The relation between physical activity and the S.V.V. precision is consistent with previous findings on postural balance [22,27,47]. Additionally, this study showed that sitting time has an independent influence on S.V.V. precision.

Physical activity is related to general health [48]. Nevertheless, physical activity counts on adequate head–body balance that requires processing of sensory inputs to attain efficient motor responses. Balance training has been used for the promotion of balance and sports-related skills; in healthy young individuals, it improves performance with moderate to large effects on both static and dynamic balance, independently from age, sex, training status, setting, and testing method [47]. Moreover, balance training is also related to systematic modulation of sensory inputs that may lead to enhancement of the capacity for motor adaptation [49]. The vestibulo-collic reflex is strengthened by regular football playing, with influence from leg/eye dominance [50]. However, exercise behavior, accounting for intensity preferences, is also related to several complex factors including personality traits, such as reactivity to environmental influences (sensory processing sensitivity) [51].

In contrast, sedentary behavior has been differentiated from insufficient physical activity by the attainment of physical activity recommendations [52]; it can be defined as any behavior characterized by energy expenditure ≤1.5 times the resting metabolic rate while in a sitting or reclining posture [53].

The association between sitting time and increased S.V.V. variability with preserved accuracy is consistent with the consequences of prolonged bed rest on balance. After prolonged bed rest, the directional precision of vestibular-evoked balance responses decreases, while the accuracy is unaffected [54]. After 60 days of bed rest, the sensorimotor transformation process that converts vestibular inputs into appropriately directed balance responses is impaired, with increased directional variability of vestibular-evoked balance responses, and increased spontaneous postural sway [54].

The sensorimotor process may be affected by inactivity, including reduced sensory information that results in maladaptive brain activation and delayed sensory reflexes [55]. A systematic review of the association between sedentary behavior and falls in old adults (n = 24 750, aged 60 to 99 y.o.) showed an increased risk of falls (odds ratio = 1.17; 95% C.I. 1.07–1.28) [56]. A five year follow-up of 308 women (36 to 56 y.o.) showed that increased sedentary time was related to decreases in functional performance [57].

### 4.3. History of COVID-19

The multivariate analysis in healthy participants also suggested contribution to S.V.V. precision from a history of COVID-19. Although the available evidence does not support that SARS-CoV-2 induces acute vestibulopathy [58], in adults with persistent COVID-19 symptoms, sensory deficits may vary according to initial disease severity, age and post-COVID cognitive dysfunctions [59]. Consistently, less accurate visual and haptic vertical perception has been observed in adults with post-COVID-19 who required hospitalization (n = 24), compared to those not hospitalized (n = 36) [60]. In this study we observed that even disease that did not require hospitalization contributed to variability among participants without diabetes in S.V.V. precision.

### 4.4. Diabetes

In this study, the major contribution of diabetes to the variability in S.V.V. precision was accompanied by a higher frequency of comorbidities and lower physical activity, compared to participants without diabetes.

In adults with diabetes or at highest risk for developing type 2 diabetes, lower levels of physical activity are frequent [61], while greater sedentary time is related to hyperglycemia, independent of aerobic fitness [62]. Additionally, several factors frequently associated with the incidence of diabetes complications can be modified by physical activity [63]. Therefore, in this study, we can presume that diabetes accounted for the contribution from physical activity and sitting time to the variability of S.V.V. precision among the participants.

Adults with diabetes have an increased risk for falls. Several factors may contribute to increase this risk, such as medication, pain, self-perceived health, cognitive impairment, lower-extremity physical performance, physical activity, and grip strength, as well as limitations in daily-life activities [64]. However, the contribution of any vestibular dysfunction is not currently understood.

In the absence of overt microangiopathy and features of peripheral neuropathy, in rats with diabetes, myelin-sheath abnormalities were found in the nerve supplies of the saccule and utricle [65]. In 39 adults with type 1/type 2 diabetes mellitus, compared to 40 age-matched adults without diabetes, the temporal bones showed no evidence of saccular alterations in arteriole thickness, but lower density of hair cells, with no difference between type 1 and type 2 diabetes [66]. In 14 adults with type 1 diabetes and 28 age-matched adults, the prevalence of cupular and free-floating deposits in the lateral and posterior semicircular canals was higher in the temporal bones of adults with diabetes [67].

The pathogenesis of diabetes is also associated with nervous system alterations, including neuroinflammation, with neurodegeneration. Abnormal regional brain activity correlates with circulating C-peptide and pancreatic β-cell function [68]. In both human and animal models, diabetes is related to microglial, astrocyte, and Müller cell increased reactivity and dysfunctionality, myelin loss, and Schwann cell alterations (review [69]); brain alterations in astrocytes, microglia, and myelin have been associated with brain atrophy, hyperpermeability of the blood–brain barrier and cognitive decline (review [69]). A meta-analysis of multimodal imaging MRI studies in adults with type 2 diabetes, compared to adults without diabetes, showed conjoint decreased regional gray matter volume, and altered intrinsic activity mainly in the bilateral superior temporal gyrus/Rolandic operculum, left temporal gyrus, and left supramarginal gyrus, as well as functional abnormalities in the cerebellum, insula, and visual cortex [70]. It has been proposed that recurrent hyper-/hypoglycemia may lead to metabolic and molecular alterations that ultimately result in cell death with atrophy [71]. Then, both peripheral and central vestibular dysfunction may lead to S.V.V. precision decrease in patients with diabetes. However, given that graviception comprises several complex processes [1,2,72,73] that can be affected by the pathophysiology of diabetes complications [68,74,75,76,77], precise characterization of the physiopathology related to vestibular dysfunction is not yet understood and requires further research.

In this study, the on-axis rotation allowed to discard peripheral otolith asymmetry that could be observed by the equal and opposite centripetal acceleration on the right and left labyrinths, while the potential contribution from the horizontal semicircular canals to the S.V.V. estimation could be considered [78]. The finding of an inconsistent contribution of the vestibular gain to sinusoidal rotation to the variability of S.V.V. precision in participants with diabetes is speculative. However, it is consistent with the evidence supporting that stimulation of the semicircular canals can influence the perception of S.V.V. S.V.V. tilts follow changes in ocular torsional position due to anticompensatory eye deviation rather than the slow phase torsional vestibulo-ocular responses [79].

### 4.5. Limitations

The results of this study should be interpreted with caution. The cross-sectional design allowed identification of associations without evidence of causality, and the study power showed the largest linear relationships without denying other potential associations or nonlinear relationships. The selection criteria limited the participation of individuals with a variety of frequent comorbidities that could modify the observed associations, implying a bias for physically active participants. However, this selection allowed to describe S.V.V. through adulthood and the major factors contributing to S.V.V. precision in adults with/without diabetes.

## 5. Conclusions

In adults without diabetes and no history of otology or vestibular disease, the S.V.V. precision but not the S.V.V. accuracy may decrease after the age of 50 years, with no further decrease after the age of 70 years (up to 80 y.o.). Cofactors contributing to S.V.V. precision variability include physical activity and sitting time; the complex pathophysiology of diabetes may account for the contribution from these cofactors. The results support that the sensorimotor process affected by both insufficient activity and sedentary behavior includes the vestibular system.

## Figures and Tables

**Table 1:** Characteristics of 262 adults without diabetes.

Variable	Women	Men	All
Number of participants	123	139	262
Years of age (mean ± S.D.)	50.0 ± 15.7	47.2 ± 15.0	48.7 ± 15.4
Body mass index (mean ± S.D.)	27.4 ± 4.2	28.8 ± 4.5	28.0 ± 4.4
Corrected refraction errors (N, %)	72 (51.7%)	81 (65.2%)	153 (58.0%)
Tobacco use (N, %)	11 (8.9%)	21 (15.1%)	32 (12.2%)
Alcohol use (N, %)	33 (23.7%)	47 (38.2%)	80 (30.0%)
History of COVID-19 (N, %)	75 (53.9%)	64 (52.0%)	139 (53.0%)
Systemic high blood pressure (N, %)	20 (14.3%)	14 (11.3%)	34 (12.9%)
Dyslipidemia (N, %)	5 (3.6%)	7 (5.6%)	12 (4.5%)
Physical activity			
Low (N, %)	8 (6.5%)	9 (6.4%)	17 (6.4%)
Moderate (N, %)	29 (23.5%)	45 (32.3%)	74 (28.2%)
High (N, %)	86 (69.9%)	85 (61.1%)	171 (65.2%)
Sitting time (hours/week) (mean ± S.D.)	31.1 ± 21.0	29.1 ± 22.5	30.0 ± 21.8

**Table 2:** Mean and Standard Deviation of the mean of the vestibular test of 262 adults without diabetes, by decade of life. Comparisons among subgroups were performed using analysis of variance. y.o. = years old. H.S.C. = Horizontal semicircular canal. S.V.V. = Subjective Visual Vertical. S.D. = Standard Deviation. d.f. = degrees of freedom.

Variable	All	21–30 y.o.	31–40 y.o.	41–50 y.o.	51–60 y.o.	61–70 y.o.	71–80 y.o.	(d.f. 255, 5)
	N = 262	(n = 45)	(n = 48)	(n = 45)	(n = 50)	(n = 50)	(n = 23)	*p* (F)
H.S.C. gain (0.16 Hz)	0.50 ± 0.14	0.55 ± 0.17	0.56 ± 0.13	0.51 ± 0.15	0.46 ± 0.12	0.47 ± 0.12	0.45 ± 0.14	0.0006 (4.49)
H.S.C. gain (1.28 Hz)	0.96 ± 0.08	0.96 ± 0.07	0.95 ± 0.07	0.95 ± 0.08	0.98 ± 0.10	0.96 ± 0.08	0.95 ± 0.09	0.65 (0.66)
Static S.V.V. (average)	0.74 ± 0.50	0.78 ± 0.45	0.83 ± 0.56	0.82 ± 0.49	0.73 ± 0.52	0.65 ± 0.47	0.59 ± 0.50	0.24 (1.35)
Static S.V.V. (S.D.)	1.13 ± 0.41	0.89 ± 0.31	0.99 ± 0.33	1.08 ± 0.38	1.25 ± 0.44	1.32 ± 0.41	1.37 ± 0.39	<0.00001 (10.19)
On-axis S.V.V. (average)	0.71 ± 0.51	0.71 ± 0.47	0.76 ± 0.48	0.60 ± 0.45	0.63 ± 0.50	0.71 ± 0.53	0.78 ± 0.64	0.53 (0.82)
On-axis S.V.V. (S.D.)	1.22 ± 0.57	1.03 ± 0.43	1.00 ± 0.44	1.23 ± 0.54	1.47 ± 0.57	1.40 ± 0.66	1.29 ± 0.64	0.00004 (5.83)

**Table 3:** Results of the multivariate analysis of covariance on the standard deviation of the Subjective Visual Vertical, during the static and on-axis conditions, of 262 adults without diabetes. d.f. = degrees of freedom.

Contributing Variables	Static Estimation	On-Axis Estimation	Multivariate
Multiple R (*p* Value)	R = 0.44 (<0.00001)	R = 0.35 (<0.00001)	(d.f. 2, 256)
	*p* (F)	*p* (F)	*p* (F)
*Intercept*	<0.00001 (47.21)	<0.00001 (46.17)	<0.00001 (38.21)
Age	<0.00001 (32.52)	0.0005 (12.31)	<0.00001 (18.89)
Physical activity	0.022 (5.26)	0.001 (11.01)	0.001 (6.77)
Sitting time	0.033 (4.57)	0.022 (5.24)	0.019 (4.02)
History of COVID-19	0.054 (3.74)	0.21 (1.54)	0.038 (3.30)

**Table 4:** Characteristics of 187 adults with diabetes and 187 adults with diabetes.

Group		Diabetes			No Diabetes	
Variable	Women	Men	All	Women	Men	All
Number of participants	86	101	187	106	81	187
Years of age (mean ± S.D.)	58.6 ± 9.3	55.6 ± 10.4	57.0 ± 10.0	58.3 ± 10.3	55.7 ± 10.7	57.2 ± 0.5
Body mass index (mean ± S.D.)	29.6 ± 4.8	28.8 ± 5.5	29.2 ± 5.0	27.8 ± 4.0	29.0 ± 4.6	28.3 ± 4.3
Years since diagnosis (mean ± S.D.)	10.5 ± 9.1	12.3 ± 8.3	11.5 ± 8.7	-	-	-
Corrected refraction errors (N, %)	20 (23.1%)	31 (30.6%)	51 (27.2%)	41 (38.6%)	46 (56.7%)	87 (45.4%)
Tobacco use (N, %)	10 (8.1%)	20 (16.2%)	30 (24.3%)	12 (11.3%)	14 (17.2%)	26 (13.9%)
Alcohol use (N, %)	10 (8.1%)	31 (30.6%)	38 (20.3%)	20 (18.8%)	25 (30.8%)	45 (24.0%)
Comorbidities						
Systemic high blood pressure (N, %)	28 (32.5%)	33 (32.6%)	61 (32.6%)	24 (0.22%)	12 (14.8%)	36 (19.2%)
History of COVID-19	39 (45.3%)	43 (42.5%)	82 (43.8%)	54 (50.9%)	38 (46.9%)	92 (49.1%)
Dyslipidemia (N, %)	16 (18.6%)	14 (13.8%)	30 (16.0%)	6 (5.7%)	6 (7.4%)	12 (6.4%)
Main complications of diabetes						
Peripheral neuropathy (N, %)	55 (63.9%)	83 (82.1%)	138 (73.7%)	-	-	-
Retinopathy (non-proliferative) (N, %)	13 (15.1%)	35 (34.6%)	48 (47.5%)	-	-	-
Physical activity						
Low (N, %)	20 (23.2%)	16 (15.8%)	36 (19.2%)	10 (9.4%)	7 (5.6%)	17 (9.0%)
Moderate (N, %)	34 (39.5%)	35 (34.6%)	69 (36.8%)	34 (32.0%)	19 (23.4%)	53 (28.3%)
High (N, %)	32 (37.2%)	50 (49.5%)	82 (43.8%)	62 (58.4%)	55 (67.9%)	117 (62.5%)
Sitting time (hours/week) (mean ± S.D.)	23.5 ± 20.2	34.1 ± 25.5	29.2 ± 23.7	23.3 ± 18.9	27.7 ± 20.2	25.2 ± 19.5

**Table 5:** Mean and Standard Deviation of the mean of the vestibular test of 187 adults with diabetes and 187 adults without diabetes. Comparisons between all the participants of each group were performed using “*t*” test. H.S.C. = Horizontal semicircular canals. S.V.V. = Subjective Visual Vertical. S.D. = Standard Deviation. d.f. = degrees of freedom.

Group		Diabetes			No Diabetes		(d.f. 372)
Variable	Women	Men	All	Women	Men	All	
	(n = 86)	(n = 101)	(n = 187)	(n = 106)	(n = 81)	(n = 187)	*p* (t)
H.S.C. gain (0.16 Hz)	0.49 ± 0.17	0.41 ± 0.14	0.44 ± 0.16	0.48 ± 0.13	0.48 ± 0.14	0.48 ± 0.14	0.016 (−2.41)
H.S.C. gain (1.28 Hz)	0.96 ± 0.08	0.97 ± 0.09	0.97 ± 0.09	0.96 ± 0.09	0.97 ± 0.08	0.96 ± 0.08	0.54 (0.61)
Static S.V.V. (average)	0.59 ± 0.53	0.78 ± 0.48	0.69 ± 0.51	0.71 ± 0.51	0.70 ± 0.47	0.71 ± 0.49	0.79 (−0.25)
Static S.V.V. (S.D.)	1.55 ± 0.59	1.38 ± 0.59	1.46 ± 0.60	1.28 ± 0.43	1.19 ± 0.40	1.24 ± 0.42	0.00004 (4.10)
On-axis S.V.V. (average)	0.68 ± 0.50	0.63 ± 0.43	0.66 ± 0.46	0.67 ± 0.54	0.73 ± 0.53	0.70 ± 0.53	0.43 (−0.78)
On-axis S.V.V. (S.D.)	1.97 ± 1.08	1.60 ± 0.84	1.77 ± 0.96	1.49 ± 0.59	1.16 ± 0.56	1.33 ± 0.61	<0.00001 (5.20)

**Table 6:** Results of the multivariate analysis of covariance on the standard deviation of the Subjective Visual Vertical on static and on-axis conditions of 187 participants with diabetes and 187 participants without diabetes.

Contributing Variables	Static Estimation	On-Axis Estimation	Multivariate
Multiple R (*p* Value)	R = 0.33 (<0.00001)	R = 0.31 (<0.00001)	(d.f. 2, 369)
	*p* (F)	*p* (F)	*p* (F)
*Intercept*	<0.00001 (21.04)	0.009 (6.73)	0.00001 (11.15)
Age	<0.00001 (22.21)	0.0008 (11.35)	<0.00001 (12.9)
Diabetes	0.00001 (19.35)	<0.00001 (28.64)	<0.00001 (18.17)
Vestibulo-ocular gain at 1.28 Hz	0.006 (7.61)	0.22 (1.45)	0.022 (3.84)

## Data Availability

All data generated or analyzed during this study are included in this article. Further enquiries can be directed to the corresponding author.

## Abbreviations

The following abbreviations are used in this manuscript:

d.f.	Degrees of Freedom
H.S.C.	Horizontal Semicircular Canal
S.V.V.	Subjective Visual Vertical
S.D.	Standard Deviation
95% C.I.	95% Confidence Interval
